# Unfolded Protein Response of the Endoplasmic Reticulum in Tumor Progression and Immunogenicity

**DOI:** 10.1155/2017/2969271

**Published:** 2017-12-21

**Authors:** Yoon Seon Yoo, Hye Gyeong Han, Young Joo Jeon

**Affiliations:** ^1^Department of Biochemistry, Chungnam National University School of Medicine, Daejeon 35015, Republic of Korea; ^2^Department of Medical Science, Chungnam National University School of Medicine, Daejeon 35015, Republic of Korea

## Abstract

The endoplasmic reticulum (ER) is a pivotal regulator of folding, quality control, trafficking, and targeting of secreted and transmembrane proteins, and accordingly, eukaryotic cells have evolved specialized machinery to ensure that the ER enables these proteins to acquire adequate folding and maturation in the presence of intrinsic and extrinsic insults. This adaptive capacity of the ER to intrinsic and extrinsic perturbations is important for maintaining protein homeostasis, which is termed proteostasis. Failure in adaptation to these perturbations leads to accumulation of misfolded or unassembled proteins in the ER, which is termed ER stress, resulting in the activation of unfolded protein response (UPR) of the ER and the execution of ER-associated degradation (ERAD) to restore homeostasis. Furthermore, both of the two axes play key roles in the control of tumor progression, inflammation, immunity, and aging. Therefore, understanding UPR of the ER and subsequent ERAD will provide new insights into the pathogenesis of many human diseases and contribute to therapeutic intervention in these diseases.

## 1. Introduction

The endoplasmic reticulum (ER) is a dynamic and specialized tubular-reticular network and extends throughout the cytoplasm in the form of connected sacs and branching tubules [1]. The ER network is heterogenous in its structure and adopts different morphologies in conjunction with different functions [2]. Interestingly, the ER is physically and functionally interconnected with every other cellular compartment and can sense intrinsic and extrinsic perturbations, combine these stress signals, and manage the cellular processes, indicating its role as a central coordinator for maintenance of cellular homeostasis [3]. The ER engages in various cellular functions involving the biosynthesis of lipid species such as cholesterol, triacylglycerol, and phospholipids, the degradation of glycogen, detoxification, and the maintenance of Ca^2+^ homeostasis [4–6]. Most importantly, the ER is involved in the synthesis, folding, maturation, and trafficking of secreted and transmembrane proteins, which constitute about one-third of all the proteins that are synthesized in the cell [5, 6]. These proteins participate in important cellular and organismal processes involving protein degradation, signal transduction, lipid metabolism, and cell-cell communications, suggesting that maintaining the integrity of these proteins is essential for life.

Protein quality control of the ER is composed of three axes, acceleration of adequate protein folding, activation of unfolded protein response (UPR), and protein clearance via ER-associated degradation (ERAD) [7]. Accumulation of misfolded and unassembled proteins can cause stress and damage, resulting in the activation of UPR to determine cell fate and function and in the subsequent restoration of protein homeostasis, which is termed proteostasis. Even with the assistance of dedicated protein folding machinery in the ER, a large portion of proteins entering the ER fails to obtain proper conformation due to mutations, unavailability of chaperones, or changes in the amounts of interacting partners and eventually must be eliminated [8, 9]. Eukaryotic cells have evolved ERAD for clearance of misfolded, unassembled, or tightly regulated proteins, resulting in the maintenance of proteostasis [10–13]. Intriguingly, a failure in the maintenance and/or restoration of the proteostasis leads to various protein misfolding diseases [14–17], implicating the importance of stringent protein quality control in the ER.

In this review, we not only discuss the molecular mechanisms of UPR of the ER and ERAD but also summarize advances in versatile aspects of these two axes. Furthermore, we provide current insights into how the adaptive capacity of the ER to intrinsic and extrinsic perturbations contributes to the modulation of malignancy, the regulation of cancer immunity, and the efficacy of therapies for cancer.

## 2. UPR of the ER

**Figure 1:**
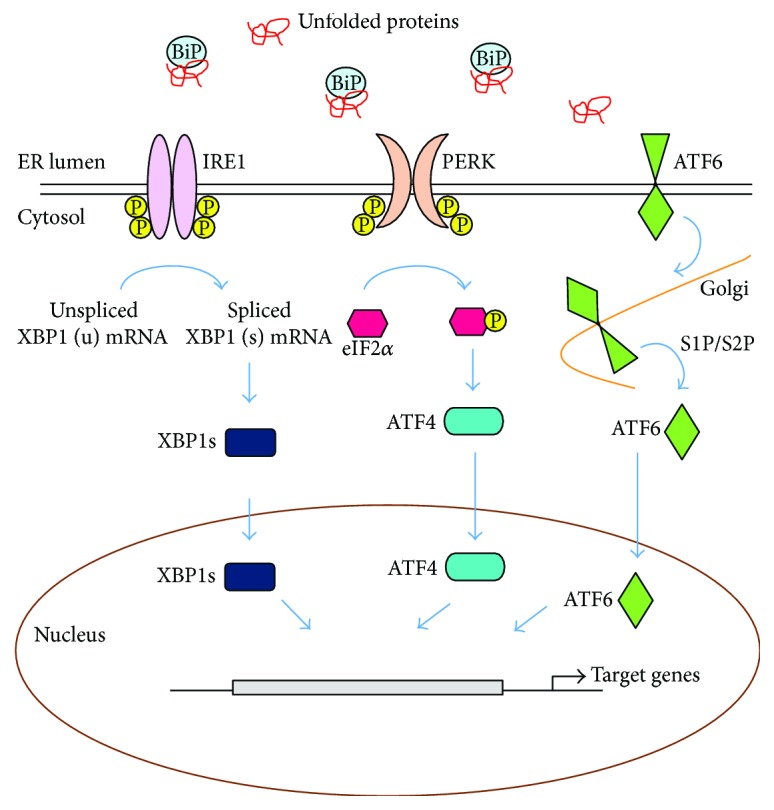
Unfolded protein response (UPR) of the endoplasmic reticulum (ER). UPR is composed of three branches of ER transmembrane sensors, IRE1, PERK, and ATF6. Upon ER stress, BiP is released from the ER sensors and is recruited to misfolded proteins, leading to the activation of UPR. Activated ER sensors transmit the stress signal into the cytosol and nucleus and subsequently operate the coordinated stress response, the UPR.

Numerous endogenous and exogenous stresses can disrupt ER protein folding environment, and unfolded or misfolded proteins accumulate in the ER, which activates UPR. While the UPR is also involved in mitochondria biology and apoptotic signal transduction, a main function of UPR is to maintain proteostasis under ER stress condition [18, 19]. In multicellular eukaryotes, UPR consists of three branches of ER transmembrane sensors, inositol-requiring protein 1 (IRE1), activating transcription factor 6 (ATF6), and protein kinase RNA- (PKR-) like ER kinase (PERK) (Figure 1). These sensors have two functional domains. The luminal domains of these sensors can sense the protein folding environment and their cytosolic domains can be connected to transcription and translation machinery. Under normal conditions, the luminal domains of these sensors are kept in an inactive state through the association with a chaperone, binding immunoglobulin protein (BiP; also known as GRP78), which belongs to the heat shock protein 70 family [20]. Upon ER stress, BiP dissociates from the ER sensors and is recruited to misfolded proteins, resulting in the activation of UPR [21–23]. It has been also known that unfolded proteins themselves can directly bind to IRE1 or PERK and this direct binding results in dimerization, oligomerization, and activation of UPR [24–27]. While the downstream response of UPR activation is a transient attenuation of global protein synthesis, an increase in a transcriptional program as well as the translation of many mRNAs including *Atf4* is induced, all of which direct towards resolving the stress [28–31]. Furthermore, when protein misfolding is not resolved, prolonged UPR activation promotes apoptosis by inducing the expression of proapoptotic genes via PERK-eIF2*α*-ATF4-CHOP axis [32, 33].

### 2.1. PERK

Upon ER stress, BiP dissociates from PERK, permitting PERK homodimerization and autophosphorylation to activate its cytoplasmic kinase domain. The activated cytosolic kinase domain of PERK in turn phosphorylates the *α* subunit of eukaryotic translation initiation factor 2 (eIF2*α*) at serine 51, which inhibits guanine nucleotide exchange factor (eIF2B) and lowers global mRNA translation, thereby attenuating the entrance of newly synthesized proteins into the ER and facilitating the cell to resolve the stress [34]. Although global mRNA translation is reduced under ER stress conditions, certain species of mRNA are favorably translated, involving activation transcription factor 4 (ATF4; also known as CREB2), which transactivates various genes, including C/EBP homologous protein (*Chop*), ER oxidoreductin 1 (*Ero1*), and growth arrest and DNA damage-inducible protein (*Gadd34*) [35–37]. Among them, CHOP is involved in ER stress-induced apoptosis under excessive and chronic activation of PERK [37, 38]. At early time points after ER stress, transcription of *Chop* is suppressed by several ways, including histone methylation and Toll-like receptor (TLR) signaling [39, 40]. However, if ER stress cannot be resolved, ATF4 and CHOP function together as a heterodimer, which increases protein synthesis, protein misfolding, oxidative stress, and finally apoptosis [17, 30].

### 2.2. IRE1

IRE1 possesses a serine/threonine kinase activity as well as endoribonuclease activity within the cytoplasmic domain [41]. Upon sensing the ER stress, IRE1 is released from BiP and activated, leading to the nonconventional splicing of a single mRNA that encodes X-box binding protein 1 (XBP1). As a result, a translational frameshift is generated and spliced *Xbp1* (XBP1s) isoform is produced [25, 42]. As a transcription factor, XBP1s induces the transcription of a wide range of targets, involving molecular chaperones and enzymes that together assist folding of polypeptides [43, 44]. In addition, XBP1s induces the expression of genes involved in membrane expansion and lipid synthesis [45]. Interestingly, once translated, an unspliced form, XBP1u negatively regulates XBP1s by promoting its proteasome-mediated degradation [46]. When ER stress persists, IRE1 is in a hyperactive state, resulting in the cleavage of many other RNAs besides *Xbp1*, involving precursors of apoptosis-inhibitory microRNAs, which in turn promotes programmed cell death [47–49].

### 2.3. ATF6

ATF6 contains a bZIP transcription factor within its cytosolic domain. Under stress-free conditions, the luminal domain of ATF6 is kept inactive via association with BiP. Upon ER stress, BiP is released from ATF6 and ATF6 is transported to the Golgi apparatus, where it is processed by the Golgi enzyme site 1 protease (S1P) and S2P, leading to the transport of its cleaved cytosolic p50 fragment into the nucleus. The cytosolic p50 fragment then induces the expression of genes such as *Xbp1* to increase the capacity of the ER to resolve ER stress as well as genes required for ERAD [50–52]. Intriguingly, XBP1s and ATF6 can heterodimerize and also induce the expression of genes involved in ERAD [52, 53].

## 3. ERAD

**Figure 2:**
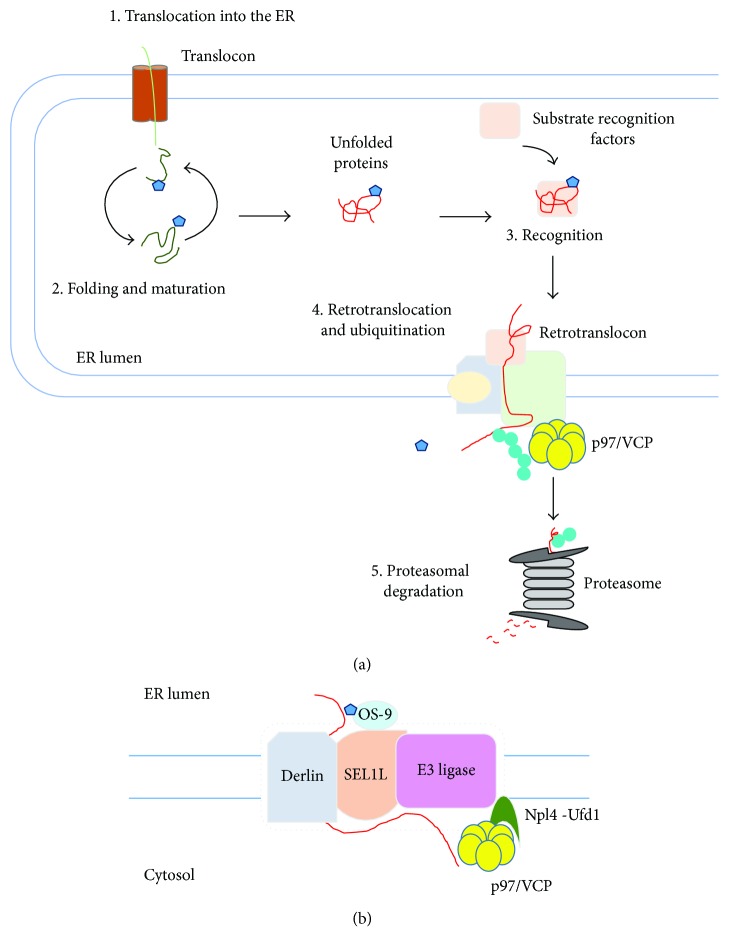
ER-associated degradation (ERAD). (a) ERAD functions to eliminate terminally misfolded, unassembled, or tightly regulated proteins by the cytosolic ubiquitin proteasome system (UPS). (1) Protein translocation into the ER through translocon. (2) Protein folding and maturation. Proteins translocated into the ER are subject to cotranslational and posttranslational folding. (3) Substrate recognition. Proteins failing to acquire their native conformation are recognized for ERAD. (4) Retrotranslocation and ubiquitination. Recognition of ERAD substrates facilitates the assembly of retrotranslocon and initiates ERAD E3 ubiquitin ligase-mediated polyubiquitination of substrates. (5) Proteasomal degradation. Carbohydrate and ubiquitin chains are removed from the retrotranslocated substrates. The retrotranslocated substrates are then inserted into the narrow channel of the proteasome, resulting in the degradation of substrates. (b) Retrotranslocation. ERAD substrate is recruited to retrotranslocon complex, which involves SEL1L, OS-9, Derlin, E3 ubiquitin ligase, and p97/Npl4/Ufd1 complex. Blue pentagon indicates *N*-glycan and green circle indicates ubiquitin.

The ER participates in the synthesis of the secretory proteins, of the luminal proteins of the ER, Golgi apparatus, endosomes, and lysosomes, and of membrane proteins. Protein synthesis in the ER is a complicated process involving targeting of ribosomes loaded with nascent polypeptide to the ER membrane, cotranslational translocation of nascent polypeptide, and co- and posttranslational folding and maturation of the polypeptide chain [2, 54]. The co- and posttranslational folding and maturation of polypeptides commence during translocation and are assisted by molecular chaperones residing in the ER [55–58]. Chaperones associate with folding intermediates, accelerate their proper folding and assembly, and prevent their improper aggregation. In addition, modifications involving *N*-linked glycosylation, proline *cis-trans* isomerization, and disulfide bond formation support proper folding of translocated polypeptides in the ER [59–62]. Nevertheless, protein maturation is not a perfect process and produces improper polypeptides, which can cause cellular stress and cytotoxicity [16, 17] and therefore must be eliminated. Eukaryotic cells have evolved ERAD to eliminate misfolded, unassembled, or metabolically regulated proteins by the cytosolic ubiquitin proteasome system (UPS) [10–13] (Figure 2). Since the late 1980s, it has been elucidated that ERAD is an elaborate and multistep process that recognizes, extracts, and ubiquitinates proteins for degradation by the cytosolic 26S proteasome [7, 13, 63–65]. In ERAD, proteins to be integrated into ER membrane or translocated into the lumen can be ultimately subject to UPS. E3 ubiquitin ligases in ERAD are spatially separated from their substrates, in part, by the ER membrane, suggesting that proofreading step is required to sort out potential ERAD targets. Polypeptides that have failed to acquire a native structure are subject to ERAD. These polypeptides are delivered to the ERAD E3 ubiquitin ligases and ubiquitinated on the cytosolic side of ER membrane. Then, the ubiquitinated substrates are subsequently extracted from the ER membrane and released into the cytoplasm for the proteasome-mediated degradation.

Recently, it is also demonstrated that ERAD plays a role in the control of degradation of some properly folded ER proteins [9]. In addition, certain viruses exploit ERAD to degrade host proteins such as major histocompatibility class I (MHC I) heavy chain and CD4 molecules, thereby escaping immune surveillance [66–68]. The human cytomegalovirus (HCMV) encodes ER membrane adaptor proteins such as US2 and US11, which bind to MHC I molecules and deliver them to ERAD [66]. Similarly, the human immunodeficiency virus- (HIV-1-) encoded adaptor protein, Vpu leads to the proteasome-mediated degradation of CD4 [67, 68]. Collectively, as a sophisticated ER protein quality control mechanism, ERAD not only functions as the gateway for the flux of proteins into the secretory pathway or membrane incorporation but also impacts intracellular organelle function and cellular communication with the extracellular environment [28]. Genetic ablation of the components involved in ERAD results in embryonic lethality in mice, indicating the importance of ERAD in the maintenance of organismal homeostasis [69–71]. A failure of the ERAD process to remove misfolded or unfolded proteins results in the accumulation of these proteins, a condition referenced as ER stress and is closely associated with a variety of human diseases, involving cancer, neurodegeneration, infectious diseases, and metabolic diseases [72].

### 3.1. Recognition

Substrate recognition must be tightly controlled, because this is the commitment step for substrate degradation in ERAD [9]. A number of proteins synthesized in the ER are cotranslationally modified by attachment of high-mannose “core” glycans, with the structure Glc_3_Man_9_GlcNAc_2_ ((Glc) glucose, (Man) mannose, (GlcNAc) *N*-acetylglucosamine), to consensus asparagine residues within canonical *N*-glycosylation sites (NxS/T) [73]. The ER quality control system uses these glycans in monitoring conformational maturation, directing correctly folded proteins to ER exit, or directing misfolded proteins to ERAD. The lectin-type chaperone, calnexin or calreticulin binds to Glc_1_Man_9_GlcNAc_2_ produced by deglucosylation of core glycans and facilitates folding of immature glycoproteins [73]. Further deglucosylation of final glucose from *N*-glycan inhibits additional binding of the glycoproteins to calnexin or calreticulin, allowing ER exit of the proteins. Interestingly, incompletely folded proteins are subject to reglucosylation by UDP-glucose : glycoprotein glucosyltransferase (UGGT). These glycoproteins reassociate with calnexin or calreticulin and undergo further rounds of folding [74, 75].

Terminally misfolded proteins must escape from calnexin/calreticulin cycle for ERAD. This escape is regulated by mannosidases that progressively remove terminal mannose residues from core glycans, permitting them to associate with mannose-specific lectins for ERAD [74, 76]. Further trimming of terminal mannoses by ER mannosidase I (ERManI) [77, 78], the ER degradation-enhancing *α*-mannosidase-like proteins 1 (EDEM1) [79, 80], EDEM3 [81, 82], or Golgi-resident mannosidase *α* class 1C member 1 (Man1C1) [83] leads to the discrimination of terminally misfolded proteins from their maturation-competent counterparts. ER-resident lectins, osteosarcoma 9 (OS-9), and XTP3-B/Erlectin then recognize these mannose-trimmed proteins through mannose-6-phosphate receptor homology (MRH) domains and recruit them to protein penetration channel, retrotranslocon [84–86]. Silencing both lectins attenuates the degradation of model substrates, while knockdown of either lectin has marginal effects in stabilizing ERAD substrates, suggesting that there may be some redundancy between them [82, 85, 87].

Whereas oligosaccharides are common for substrate recognition step, features besides glycan trimming can contribute to the targeting of folding-defective proteins to ERAD. The nonlectin chaperone BiP associates with glycoproteins as well as nonglycosylated proteins for targeting to ERAD [88, 89]. In addition, EDEM1 is involved in targeting of unglycosylated proteins to ERAD [90]. Redox-driven protein disulfide isomerase (PDI) that is characterized by thioredoxin-like motifs [91] is also involved in ERAD [92]. Interaction of chaperones with ERAD substrates permits the association of substrates with PDI.

### 3.2. Retrotranslocation

Energy-dependent protein extraction across the ER membrane back into the cytoplasm is a step known as dislocation or retrotranslocation [93] (Figure 2(b)). Importantly, no evidence indicates that the ER lumen contains any components involved in UPS such as E1 ubiquitin-activating enzyme, E2 ubiquitin-conjugating enzyme, or the proteasome, implicating that retrotranslocation is an essential step for degradation of ERAD substrates. Intriguingly, the processes of retrotranslocation and proteasomal degradation should be also tightly coupled, because many ERAD substrates are highly hydrophobic and easily aggregate in an aqueous environment. Therefore, a number of adaptors that recognize a diverse set of features through which substrates are committed to ERAD are essential for recruitment of ERAD substrates to retrotranslocons. As one of the most abundant proteins, p97/valosin-containing protein (VCP), a homohexameric enzyme, is a member of the type II AAA+ protein family of ATPases and consists of two AAA domains, D1 and D2, that are assembled in a head-to-tail manner, as well as an N-terminal domain that plays a role in substrate recognition [94–97]. In addition, the C-terminal domain of p97/VCP associates with a large number of adaptors, explaining the diversity of p97/VCP interacting partners [96]. p97/VCP has been demonstrated to be implicated in chromatin remodeling, autophagosome maturation, proteasome-mediated degradation, and ER membrane fusion [98, 99]. Importantly, p97/VCP is crucial for the clearance of misfolded proteins by affecting a large number of protein homeostatic mechanisms. p97/VCP couples ATP hydrolysis to unfolding of ERAD substrates and functions in the retrotranslocation of nearly all ERAD substrates, along with cofactors recruited through p97/VCP-binding domains, involving VIM, VBR, and SHP [94].

Several studies suggest that Derlins are part of the retrotranslocon channel [100, 101]. Mammalian cells have three Derlins, Derlin-1, Derlin-2, and Derlin-3. As a rhomboid-like protein, Derlin-1 has six membrane-spanning domains and homo- or heterooligomerizes with Derlin-2 and Derlin-3 [102–107]. Derlins are related to rhomboid proteases such as ER-resident intramembrane protein RHBDL4, which cleaves unstable single-membrane-spanning or polytopic membrane proteins [108]. However, Derlins are deficient in proteolytic activity, implicating that these proteins associate with ERAD substrates and target them to p97/VCP for retrotranslocation and to E3 ubiquitin ligases for ubiquitination [109].

Suppressor/enhancer of Lin12-like (SEL1L) recruits luminal substrate recognition factors, involving OS-9, XTP3-B, EDEMs, ERdj5, and PDI to components of the retrotranslocon [64, 110]. In addition, SEL1L serves as a scaffold for the formation of a complex with integral membrane ERAD components that include Derlin-1, Derlin-2, ancient ubiquitous protein 1 (AUP1), ubiquitin regulatory X (UBX) domain-containing protein 8 (UBXD8), and VCP-interacting membrane protein (VIMP) [85, 86, 111–115], which in turn recruits the p97/VCP, thereby leading to substrate retrotranslocation. Furthermore, SEL1L is not only required for the transfer of substrates from ER lectins to E3 ubiquitin ligase hydroxymethylglutaryl reductase degradation protein 1 (HRD1) but also crucial for the stabilization of HRD1, suggesting that SEL1L is important for ERAD substrate recruitment, retrotranslocation, and ubiquitination [85, 116–120].

Erlin1/2, heterotetrameric complex located in the ER membrane rapidly associates with inositol 1,4,5-triphosphate receptors (IP_3_R) for activation of IP_3_R and links IP_3_R to the ER-resident E3 ubiquitin ligase RNF170, indicating its role in the degradation of membrane-integrated substrates [121].

### 3.3. Ubiquitination

Ubiquitination is a reversible process that conjugates ubiquitin to target proteins, which in most, but not all, cases leads to proteasome-mediated degradation of ubiquitinated proteins and is conserved in all eukaryotes. Ubiquitin is covalently attached to target proteins by a sequential enzymatic system consisting of E1 ubiquitin-activating, E2 ubiquitin-conjugating, and E3 ubiquitin-ligating enzymes [13, 122]. Additionally, removal of ubiquitin catalyzed by deubiquitinating enzymes also plays key roles in the control of numerous biological pathways [123]. In the initial step of ubiquitination, an E1 ubiquitin-activating enzyme activates ubiquitin and forms a thioester bond with ubiquitin. In the next step, an E2 ubiquitin-conjugating enzyme transfers the ubiquitin from the E1 to the target protein, which is assisted by an E3 ubiquitin ligase. Ubiquitin is normally conjugated via its C-terminus to lysine or, in some cases, to serine, threonine, or cysteine residues on the target proteins [63, 124–126]. Once ubiquitinated, ubiquitin can be further extended by the additional ubiquitin moieties on one of the lysine residues within ubiquitin, involving K6, K11, K27, K29, K33, K48, and K63 or its N-terminus [127–130]. The linkages of ubiquitin chains confer diverse structural properties to ubiquitin chains, providing a different binding platform for various processes.

In mammalian cells, more than a dozen E3 ubiquitin ligases have been demonstrated to be involved in ERAD. Several ERAD E3 ubiquitin ligases are transmembrane proteins, involving HRD1, glycoprotein 78 (gp78), membrane-associated RING (really interesting new gene) finger protein 6 (MARCH6), and RNF5 [63, 131–138]. In addition, cytoplasmic E3 ubiquitin ligases involving parkin, CHIP, SCF complexes with the F-box proteins Fbx2, Fbx6, and *β*-TrCP1/2, Smurf1, and Nrdp1/FLRF have been demonstrated to be involved in ERAD [139–145]. ERAD E3 ubiquitin ligases accomplish ERAD substrate processing in parallel with multiple E3 ubiquitin ligases, by conjugating ubiquitin to different sites of a substrate at the same time, by an initial monoubiquitination and extension by E4 ubiquitin ligase, or via sequential rounds of ubiquitination and deubiquitination, suggesting that various strategies have been evolved for optimal efficiency of ERAD [136, 146, 147]. For instance, gp78 and Trc8 cooperate as E3 ubiquitin ligase pairs to degrade HMG-CoAR [146]. RNF5 functions sequentially with CHIP to degrade misfolded CFTRΔF508 and also serves as a primer for gp78-mediated chain elongation [135, 136]. In addition to the ubiquitination of ERAD substrates, ERAD E3 ubiquitin ligases may ubiquitinate other ERAD components to recruit p97/VCP or other ERAD components that possess ubiquitin-binding domains (UBDs), involving gp78, AUP1, ubiquitin-associated- (UBA-) domain-containing protein 2 (UBAC2), and UBX domain-containing protein 8 (UBXD8) [63, 148, 149]. Interestingly, ERAD E3 ubiquitin ligases ubiquitinate each other and form a negative feedback loop, thereby leading to fine-tuning of ERAD [64, 136, 146, 150, 151].

The ER membrane-embedded E3 ubiquitin ligases comprise a part of the retrotranslocon, and inhibiting ubiquitination attenuates retrotranslocation of ERAD substrates, suggesting that retrotranslocation is tightly coupled with ubiquitination [109]. Derlin-1 and Derlin-2 are closely linked to E3 ubiquitin ligases such as HRD1, gp78, and RNF5 to form huge complexes spanning the ER membrane [103, 104, 115, 135, 152–154]. Additionally, it is speculated that Hrd1p in yeast functions as an essential part of retrotranslocon with its cofactors, thereby recruiting ERAD substrates and in turn promoting their retrotranslocation from the ER [155].

Most of the p97/VCP cofactors possess UBDs and associate directly with ubiquitinated substrates. p97/VCP and its cofactors, Npl4 and Ufd1, cooperatively produce a driving force for the retrotranslocation of ERAD substrates [156, 157]. The ERAD substrate is slightly exposed to the ER surface through the retrotranslocon, which in turn is subject to E3 ubiquitin ligase-mediated polyubiquitination, and further retrotranslocated by the p97/Npl4/Ufd1 complex, which can recognize the polyubiquitinated substrate, suggesting that polyubiquitination serves as a binding site that promotes p97/VCP-mediated substrate extraction. To summarize, membrane-embedded ERAD components such as UBXD2, UBXD8, and VIMP, ERAD E3 ubiquitin ligases, such as gp78 and HRD1, and Derlins have p97/VCP-binding motifs, implicating that p97/VCP provides a platform for these factors to regulate ubiquitination at the sites of retrotranslocation [63, 102, 104, 158–160].

### 3.4. Proteasome-Mediated Degradation

p97/VCP is also closely linked to proteasome-mediated degradation of ERAD substrates [94]. p97/VCP plays a key role in linking retrotranslocated substrates to cytoplasmic cofactors involved in further processing of substrates. The deglycosylating enzyme NGly1 localized in the cytoplasm is recruited to retrotranslocon complexes through direct binding to p97/VCP and cleaves *N*-linked glycans from retrotranslocated ERAD substrates [161, 162]. In addition, deubiquitinating enzymes (DUBs), involving YOD1 (OTUD2), VCIP135, USP13, and Ataxin-3, associate with p97/VCP either directly or indirectly and are implicated in ERAD [163–165]. Recently, it is demonstrated that impairment of p97/VCP-associated deubiquitination or expression of dominant-negative YOD1 attenuates retrotranslocation and degradation of ERAD substrates, whereas expression of p97/VCP-associating DUB restores them [163], indicating that sequential rounds of ubiquitination and deubiquitination are essential for efficient ERAD process.

Retrotranslocated substrates need to be rapidly degraded to prevent misfolded proteins from aggregating in the cytoplasm. A chaperone complex consisting of Bag6-Ubl4A-Trc35 and a cochaperone SGTA is involved in this process. Bag6 is a cytosolic chaperone and forms a large homooligomer through proline-rich domain. The proline-rich domain is sufficient for binding to the hydrophobic segments of misfolded proteins and maintaining them in a soluble state [166]. The holdase activity of Bag6 is required to maintain some retrotranslocated substrates in a competent state for proteasome-mediated degradation [167]. Ubl4A, an adaptor of Bag6, associates with SGTA via its noncanonical ubiquitin-like (UBL) domain [168]. Bag6 also associates with proteasome and adaptor proteins of proteasome, suggesting that Bag6 transfers retrotranslocated substrates to proteasome for degradation.

## 4. UPR of the ER and Cancer

UPR of the ER has been demonstrated in diverse human cancers. In fact, it has been documented that UPR of the ER plays a crucial role in the control of tumor progression and affects tumor microenvironment involving immune cells and endothelial cells [6]. UPR of the ER modulates the expression and/or the function of oncogenes or tumor-suppressive genes in cancer, which leads to an increase in protein synthesis, resulting in an increased necessity of protein-folding capacity of the ER and subsequent activation of UPR to improve the adaptive capacity of the ER. However, persistent activation of UPR consequently affects cancer cell survival, metastasis, angiogenesis, immunogenicity, and drug resistance [6].

### 4.1. UPR of the ER and Tumorigenesis

During malignant transformation, tumor cells are exposed to not only extrinsic stresses such as nutrient deprivation, accumulation of acidic waste, and hypoxia but also intrinsic stresses such as alteration in chromosome number, activation of oncogenes, inactivation of tumor-suppressive genes, and accelerated secretion, thereby triggering exacerbated protein synthesis, which results in a cellular state of ER stress and subsequently activates UPR of the ER [169–172]. It is also suggested that chronic UPR at later stages leads to adaptation of tumor to extrinsic and intrinsic perturbations and confers resistance to ER stress-induced apoptosis on tumor, while transient UPR at early stages of tumorigenesis often impedes tumor progression [173].

Oncogenic RAS-mediated transformation of melanocytes activates UPR, which induces cell cycle arrest coupled with vacuolization and ER expansion, resulting in premature senescence [174]. In models of RET-induced fibroblast transformation, UPR activation plays a protective role against oncogene-induced malignant progression through the proapoptotic CHOP pathway [175]. In a model of KRAS-transformed lung tumor, high caloric diet-induced ER stress hinders tumor growth [176]. In addition, depletion of XBP1s is known to promote tumorigenesis, suggesting that UPR of the ER may play a tumor-suppressive role [177].

PERK has been described in the initiation and progression of various tumors. Depletion of PERK leads to tumor progression [178, 179]. ATF4-CHOP axis in PERK pathway promotes protein synthesis and in turn accelerates ROS production from oxidative protein folding in the ER. The treatment of antioxidant and depletion of RPL24 reduced apoptosis by decreasing ROS production and protein synthesis, indicating that PERK is involved in tumor regression [30]. In contrast, PERK facilitates tumor growth through the stabilization of NRF2, the modulation of redox homeostasis as well as of metabolism, and the regulation of lipid biosynthesis [178, 180–184]. Intriguingly, eIF2*α* phosphorylation by PERK facilitates LC3 lipidation, autophagy initiation, and subsequent survival [185]. Additionally, it is also demonstrated that ATF4-CHOP axis induces the expression of numerous genes involved in autophagophore formation and maturation, including *Atg5*, *Atg12*, *Atg16l1*, and *Becn1* [186].

IRE1 is also involved in tumor progression. JNK activation by IRE1 suppresses antiapoptotic BCL2 activity and accelerates the action of proapoptotic BIM, leading to cell death [6]. In addition, IRE1-dependent decay of mRNA (RIDD) activates proapoptotic caspase-2 in MEFs and facilitates the expression of gene encoding thioredoxin-interacting protein (*Txnip*) in pancreatic *β* cells [48, 49]. On the contrary, IRE1-mediated activation of STAT3 and NF-*κ*B upregulates the expression of antiapoptotic proteins, involving BCL2 family members, caspase-8 inhibitor c-FLIP, MCL1, and inhibitor of apoptosis protein (IAP) [187]. IRE1-XBP1 axis is also demonstrated to correlate with poor prognosis in glioblastoma and pre-B acute lymphoblastic leukemia [188–192]. Additionally, mutated forms of IRE1 facilitate tumor progression, although some of these mutants have intact kinase and endoribonuclease activity [47, 193, 194].

ATF6-dependent p58 (IPK) restricts apoptosis during oncogenic transformation via the inhibition of PERK [175]. ATF6 also facilitates the survival of glucose restriction-resistant squamous carcinoma cells [195].

In order to provide sufficient oxygen and nutrients, growing cancer cells produce proangiogenic factors to initiate vascularization. Several studies indicate that UPR facilitates angiogenesis. PERK upregulates the expression of the vessel growth and stabilization factors VCIP and PDGFRB [179]. In addition, PERK-mediated upregulation of fibroblast growth factor 2 (FGF2), vascular endothelial growth factor (VEGF), and interleukin-6 (IL-6) and downregulation of antiangiogenic cytokines remarkably promote tumor growth and vascularization [196]. IRE1-XBP1 axis also facilitates angiogenesis via the association of XBP1s with hypoxia-inducing factor 1*α* (HIF1*α*), a key regulator of VEGF in triple negative breast cancer (TNBC) cells [197]. Intriguingly, VEGF signaling also activates UPR in endothelial cells through a phospholipase C*γ*-mTORC1 pathway, indicating that VEGF signaling and UPR may operate a positive feedback loop for angiogenesis [198].

UPR of the ER has begun to be elucidated in metastasis. Metastasis is a complicated process in which cancer cells migrate from the original tumor site, infiltrate extracellular matrix (ECM) and stromal cell layers, penetrate the lymphatic circulatory systems, colonize foreign tissues, and grow into new tumor mass [172, 199–201]. PERK-ATF4 axis activates lysosome-associated membrane protein 3 (LAMP3), thereby facilitating metastasis of hypoxic breast cancer cells [202, 203]. The upregulation of ATF4 in esophageal squamous carcinoma leads to an increase in metastasis through the regulation of matrix metalloproteinases [204]. Intriguingly, ATF4-mediated gene expression is potentially correlated with the expression of genes involved in epithelial-to-mesenchymal transition (EMT) [199].

IRE1-XBP1 axis is also implicated in metastasis. TNBC cell lines constitutively express XBP1s, and silencing of *Xbp1* potentially inhibits metastasis [197]. XBP1s drives TNBC tumorigenicity and invasiveness by assembling a transcriptional complex with HIF1*α* that upregulates the expression of HIF1*α* targets such as PDK1 and GLUT1. On the contrary, while inhibition of IRE1 in malignant glioma correlates with the downregulation of proangiogenic factors such as VEGF-A, IL-1*β*, IL-6, and IL-8, it induces a significant upregulation of proteins linked to mesenchymal differentiation and glioma invasiveness such as SPARC, decorin, and thrombospondin-1, demonstrating that IRE1 in malignant glioma promotes the formation of functional tumor blood vessels and attenuates tumor cell invasion as well as vessel cooption [205, 206]. Therefore, a comprehensive analysis of IRE1-XBP1 axis is required to determine the relationship between invasiveness and angiogenesis. Additionally, the different consequences of UPR activation likely result from an interplay between particular axes of signaling pathways within specific tumor contexts.

### 4.2. UPR of the ER and Cancer Immunogenicity

It is of great importance to explore the crosstalk between UPR of the ER in tumor cells, the release of damage-associated molecules, and the activation of immune responses for the understanding of anti-tumor immunity. Through a process “transmissible ER stress,” ER stress enables cancer cells to secrete some factors that promote macrophage activation and induce a proinflammatory response in the microenvironment of tumors [207]. This process represses the antigen-presenting capacity of bone-marrow-derived dendritic cells (DCs) and inhibits T cell proliferation, which promotes the upregulation of immunosuppressive molecules [208], suggesting that ER stress signaling may facilitate immune escape. On the contrary, ER stress also triggers immunogenic cell death (ICD) and antitumor immunity [209]. The ICD provokes release of damage-associated molecular patterns (DAMPs), involving surface exposure of calreticulin, ATP secretion, and passive release of high-mobility group box 1 (HMGB1), suggesting that DAMPs serve as signals of danger and facilitate antitumor immunity [173, 210, 211]. PERK-eIF2*α* axis is associated with the exposure of calreticulin in non-small-cell lung carcinoma (NSCLC) and is correlated with ICD and antitumor immunity [212]. Photodynamic therapy increases the surface exposure of calreticulin as well as ATP secretion via PERK signaling in human bladder carcinoma, leading to engulfment of cancer cells by dendritic cells (DCs) [213]. In addition, radiation and anthracycline treatment induce lethal ER stress characterized by ROS production, an increase in the level of cytosolic Ca^2+^, and the excessive activation of UPR, thereby leading to the activation of inflammasome and subsequent ICD [48, 214]. However, IRE1-XBP1 axis is demonstrated to prevent the induction of ICD in metastatic colorectal cancer cells exposed to chemotherapy [215].

The tumor microenvironment is a complex environment consisting of stromal cells such as fibroblasts and endothelial cells and infiltrating immune cells such as CD8 T cells, Tregs, myeloid-derived suppressor cells (MDSCs), and DCs. Recently, it has begun to emerge as a new research area to elucidate the relationship between ER stress response in tumor-associated immune cells and tumor progression [199]. IRE1-XBP1 axis is essential for the differentiation of plasma cells and some dendritic cells [216–218]. ER stress response driven by XBP1 hyperactivation promotes neutrophil-infiltrating acute lung injury [219]. Additionally, XBP1 is required for the production of IL-6 in macrophages [220]. Persistent activation of IRE1-XBP1 axis is found in ovarian tumor-infiltrating DCs [221]. Intriguingly, the ovarian tumor-infiltrating DCs facilitate ROS production and consequential disruption of ER homeostasis, thereby leading to the control of antitumor immunity. In addition, the status of ROS-promoted lipid peroxidation has been suggested as a biomarker of disease recurrence in breast cancer patients [222]. Consistently, tumor-infiltrating DCs lacking XBP1 acquire immunostimulatory and antitumoral characteristics *in vivo* [223–225]. Pharmacological inhibition of IRE1 in bone-marrow-derived macrophages stimulated by IL-6 and IL-4 attenuates macrophage-mediated cell invasion *in vitro* [226]. Interestingly, IL-4 and IL-6 synergistically activate IRE1-XBP1 axis in macrophages. In addition, pharmacological induction of ER stress triggers the upregulation of the lectin-type oxidized LDL receptor-1 (LOX-1) in neutrophils and can induce transformation of neutrophils into immunosuppressive cells [227, 228]. These studies suggest that IRE1-XBP1 axis plays a key role in the control of tumor-associated myeloid cells.

CHOP is known to be upregulated in tumor-infiltrating MDSCs [229]. Tumor-infiltrating MDSCs devoid of CHOP show reduced immunosuppressive activity toward T cells due to defective expression of arginase. In addition, UPR activation in tumor-infiltrating MDSCs promotes apoptosis through death receptor 5 (DR5) and caspase-8 activation [230]. To summarize, these findings suggest that UPR activation plays a pivotal role in fine-tuning of tumor-associated immune responses.

### 4.3. UPR of the ER and Therapies for Cancer

UPR-activating or inhibiting strategies have begun to emerge as new pharmacological tools for cancer treatment. A large number of anticancer drugs induce UPR activation and facilitate the development of chemosensitivity or chemoresistance in a context-dependent manner [199]. Anticancer drugs involving paclitaxel, the epidermal growth factor receptor (EGFR) inhibitor cetuximab, and the BRAF (V600E) inhibitor vemurafenib induce PERK-mediated eIF2α phosphorylation [137, 215, 231]. In addition, nonsteroidal anti-inflammatory drugs promote UPR-mediated apoptosis and are used in combined anticancer therapies [232, 233].

Recently, targeting of UPR of the ER in cancer cells has been demonstrated to inhibit survival or promote cell death [173, 234, 235]. Therapies targeting UPR are applied for multiple myeloma or B cell-associated hematologic malignancies [173]. In addition, inhibition of PERK-eIF2*α* axis promotes cell death of therapy-resistant hypoxic glioblastoma and colon carcinoma cells [236], suggesting that combination of cancer therapy and UPR targeting may be desirable for cancer treatment.

ER stress-induced autophagy may facilitate therapeutic resistance. Autophagy induced by IRE1-JNK pathway facilitates sorafenib resistance in hepatocellular carcinoma cell lines [237, 238]. It is also demonstrated that a marked increase in cytoprotective autophagy induced by PERK develops vemurafenib resistance in melanoma [231]. Intriguingly, simultaneous inhibition of BRAF (V600E) and PERK sensitizes chemoresistant melanoma to ER stress-induced apoptosis, suggesting that the balance between autophagy mediated by UPR and chemotherapy is required for the overcome of chemoresistance.

Because ER stress modulates the functions of tumor-associated immune cells and subsequently protumoral or antitumor immune responses, it is noteworthy to consider UPR-targeting therapies in immune cells. Targeting IRE1-XBP axis in DCs has been demonstrated to be effective for cancer treatment [221, 239, 240]. Depletion of Xbp1 or silencing of *Ire1* in preclinical models remarkably transforms DCs into immunostimulatory cells, thereby promoting survival through T-cell-mediated antitumor immunity [221]. In addition, transplanted MDSCs devoid of DNA damage-inducible transcript 3 (*Ddit3*) show enhanced antigen-presenting capacity and T cell stimulatory effects [229]. Intriguingly, ERO1*α* is demonstrated to upregulate programmed death ligand 1 (PDL1) in TNBCs [241]. These studies suggest that the combination of UPR-modulating therapies with immunotherapies might be effective for cancer treatment.

## 5. ERAD and Cancer

ERAD in tumor progression and immunogenicity is not well known. Several studies suggest that the high degree of cell division and high mutation rates in cancer cells lead to an accumulation of misfolded proteins, which activates ERAD [242, 243]. Higher expression of SEL1L in pancreatic cancer cells leads to not only G1 phase cell cycle arrest via the induction of a phosphatase and tensin homolog (PTEN) but also reduction in invasiveness by modulating genes related to cell-matrix interactions [244, 245]. Additionally, low expression of SEL1L in breast cancer patients has been reported to correlate with poor prognosis [246]. However, in the context of colorectal cancer, while basal expression of SEL1L in normal mucosa of the epithelial lining is low, SEL1L is upregulated in adenoma and adenocarcinoma cells [247]. Therefore, it is pivotal to elucidate the mechanism by which SEL1L is involved in tumor progression within a specific tumor context and the effects of changes in SEL1L expression in tumor cells on ERAD substrates or ER homeostasis to clarify the role of SEL1L in cancer pathogenesis.

Under hypoxic conditions, OS-9-mediated degradation of HIF1*α* is crucial in the downregulation of genes that promote cell survival, proliferation, angiogenesis, and metastasis [248–250], suggesting that OS-9 is important in the regulation of tumor progression.

gp78 induces a signaling cascade to mediate tumorigenesis and is linked with various types of cancers [251–253]. gp78 is highly expressed in bladder carcinoma tissues, and colorectal cancer patients with higher expression of gp78 have less survival and high risk of cancer recurrence, suggesting that gp78 is closely related to increased risk of cancer with lower survival rate [254–256].

The role of gp78 in metastasis is largely unknown. However, several studies suggest the involvement of gp78 in metastasis. Expression of gp78 is modulated by cell-cell contact, and loss of this balance is linked to metastasis [257]. Intriguingly, an inverse correlation between gp78 and E-cadherin has been reported in patients with bladder carcinomas as well as gastric cancers [258–260]. gp78-mediated degradation of metastasis suppressor protein Kangai1 (KAI1) promotes metastasis [261, 262]. Additionally, gp78 activates ROCK2, an important metastasis-associated protein, indicating the involvement of gp78 in metastasis [263].

CHIP, cytosolic E3 ubiquitin ligase involved in ERAD is inversely correlated with malignancy in breast cancers and depletion of CHIP results in an increase in the growth of subcutaneous tumors, indicating its role as a tumor suppressor [264].

## 6. Conclusion

Organisms are continuously exposed to extrinsic and intrinsic stresses that destroy proteostasis and subsequently result in protein misfolding and aggregation, thereby leading to the state of ER stress. To restore proteostasis, eukaryotic cells have evolved UPR of the ER and ERAD as key adaptive responses. Intriguingly, a failure in these adaptive responses leads to various protein misfolding diseases, involving cancer. UPR of the ER not only acts as a guardian of tumor progression at an early stage but also serves as a key player for maintenance of tumors under chronic ER stress. Additionally, UPR has been described to manipulate immune cells in tumor microenvironment, resulting in antitumor immunity or immune escape. Importantly, UPR activation also develops chemosensitivity or chemoresistance in a context-dependent manner. Overall, UPR of the ER is involved in the modulation of tumor growth, metastasis, and angiogenesis; the interaction of tumor and stromal cells; and the regulation of inflammatory/immune responses. Therefore, to elucidate the precise molecular mechanisms by which UPR of the ER and ERAD coordinate tumor progression at different stages and modulate the communication between tumor and tumor microenvironment remarkably contributes to novel therapeutic interventions.
